# Fused Traditional and Geometric Morphometrics Demonstrate Pinniped Whisker Diversity

**DOI:** 10.1371/journal.pone.0034481

**Published:** 2012-04-03

**Authors:** Carly C. Ginter, Thomas J. DeWitt, Frank E. Fish, Christopher D. Marshall

**Affiliations:** 1 Department of Wildlife and Fisheries Sciences, Texas A&M University, College Station, Texas, United States of America; 2 Department of Biology, West Chester University, West Chester, Pennsylvania, United States of America; 3 Department of Marine Biology, Texas A&M University, Galveston, Texas, United States of America; The University of Plymouth, United Kingdom

## Abstract

Vibrissae (whiskers) are important components of the mammalian tactile sensory system, and primarily function as detectors of vibrotactile information from the environment. Pinnipeds possess the largest vibrissae among mammals and their vibrissal hair shafts demonstrate a diversity of shapes. The vibrissae of most phocid seals exhibit a beaded morphology with repeating sequences of crests and troughs along their length. However, there are few detailed analyses of pinniped vibrissal morphology, and these are limited to a few species. Therefore, we comparatively characterized differences in vibrissal hair shaft morphologies among phocid species with a beaded profile, phocid species with a smooth profile, and otariids with a smooth profile using traditional and geometric morphometric methods. Traditional morphometric measurements (peak-to-peak distance, crest width, trough width and total length) were collected using digital photographs. Elliptic Fourier analysis (geometric morphometrics) was used to quantify the outlines of whole vibrissae. The traditional and geometric morphometric datasets were subsequently combined by mathematically scaling each to true rank, followed by a single eigendecomposition. Quadratic discriminant function analysis demonstrated that 79.3, 97.8 and 100% of individuals could be correctly classified to their species based on vibrissal shape variables in the traditional, geometric and combined morphometric analyses, respectively. Phocids with beaded vibrissae, phocids with smooth vibrissae, and otariids each occupied distinct morphospace in the geometric morphometric and combined data analyses. Otariids split into two groups in the geometric morphometric analysis and gray seals appeared intermediate between beaded- and smooth-whiskered species in the traditional and combined analyses. Vibrissal hair shafts modulate the transduction of environmental stimuli to the mechanoreceptors in the follicle-sinus complex (F-SC), which results in vibrotactile reception, but it is currently unclear how the diversity of shapes affects environmental signal modulation.

## Introduction

Many organisms possess highly sensitive mechanosensory structures to monitor and detect physical cues in their environment. Mammalian vibrissae (whiskers) are finely tuned sensory structures. The vibrissae include a follicle-sinus complex (F-SC) with numerous and various types of mechanoreceptors within its complex microstructure and a vibrissal hair shaft that transmits vibrotactile environmental stimuli to these mechanoreceptors deep in the F-SC [1]–[4]. Although most mammals possess vibrissae, the majority of our knowledge regarding their function is limited to laboratory animals [5]–[8]. The number, geometric arrangement, size, morphology, shape and stiffness of vibrissae vary widely among mammals [9]. Pinnipeds (seals, sea lions and walruses) possess the largest vibrissae among mammals (e.g., Antarctic fur seals (*Arctocephalus gazella*) have vibrissae up to 480 mm long; [10]) and exhibit a diversity of shapes in these structures [9], [11], [12]. In particular, the mystacial vibrissae of phocid seals, with the exception of bearded (*Erignathus barbatus*) and monk (*Monachus* spp.) seals, show a repeating sequence of crests and troughs along their length, giving them a beaded appearance [11]–[17].

Many mammals, terrestrial and aquatic, use vibrissae for active touch and other discrimination tasks. However, no other mammal exhibits the unusual beaded vibrissal morphology possessed by most phocid seals. Since vision is a limited sensory modality in aquatic habitats, it follows that marine mammals may have experienced strong selection for compensatory sensory adaptations that facilitate functions such as prey detection, particularly when foraging at night, in turbid water or when diving deeply. Whereas odontocetes (toothed whales) evolved echolocation, pinnipeds have highly derived vibrissal sensory systems [14], [18]–[21]. Reports of healthy but blind seals foraging successfully in the wild suggest the importance of this sensory system for the aquatic environment [15], [22]. Experimental evidence has shown that phocid seals rely heavily upon their vibrissae to follow hydrodynamic trails [19], [20], [23]–[25], as well as to orient themselves when vision is restricted [26]. Vibrissae are not shed with the rest of the pelage in an annual molt, but rather individually and irregularly throughout the year [9], [27]. The shortest vibrissae on the muzzles of phocid species have been observed not to have beads but the development of beading is not currently understood [17]. Stable isotope studies of vibrissal growth rates found that otariids retain their vibrissae for over two years, while phocid vibrissae appear to grow more quickly and be replaced more often [28], [29]. The periodic and intermittent shedding and re-growth of vibrissae likely serves to avoid a decrease in sensory capability if all vibrissae were shed simultaneously or due to vibrissae loss or physical damage [9], [28], [29]. The divergence of the vibrissae from the shedding cycle of the pelage underscores the functional significance of this sensory system.

The distinctive shape of phocid seal vibrissae, with a sinusoidal beaded profile, has been shown to decrease vibrations during ambient flow while the seal is swimming, compared to smooth vibrissae [30]. Previous morphological analyses of phocid vibrissae demonstrated species-specific differences [17]. However, there are few quantitative data regarding the morphology of phocid vibrissae and, to our knowledge, no characterization of their geometry has been conducted. Therefore, we conducted a comparative study on the shape and morphology of pinniped vibrissae. We tested the hypothesis that the beaded morphology of phocid vibrissae is conserved with variants in peak-to-peak distance and crest and trough width representing species-specific differences. We also hypothesized that phocids with beaded vibrissae, phocids with smooth vibrissae and otariids would each occupy distinct morphospace from each other, which may facilitate functional differences with ecological consequences.

## Methods

### Ethics Statement

All samples were collected under a National Marine Fisheries Service (NMFS) Southeast Regional Office salvage permit letter to CDM and NMFS permits #358-1585 and 358-1787 issued to the Alaska Department of Fish and Game.

Mystacial vibrissal hair shafts external to the follicle (hereafter simply termed vibrissae) from 11 pinniped species and 92 individuals were analyzed to quantify shape and morphological differences among species with beaded and smooth vibrissal profiles (Table 1). Samples were collected from dead, stranded animals in New Jersey, New England and California, from legal indigenous hunts in Alaska and opportunistically when shed by captive animals. To standardize our comparisons, the longest vibrissae which did not show any wear or breakage from each individual were used. These vibrissae always occurred in the most lateral portions of the lower rows of the mystacial vibrissal field. Scaled digital photographs (27.0 pixels/mm) of whole vibrissae were taken with a Nikon D200 SLR camera. To maximize the contrast between the background and the vibrissa in the photograph, all vibrissae were dyed black (using Revlon ColorSilk hair dye #10; Revlon Cons. Prod. Corp., N.Y., NY 10017). Vibrissae were coated with the dye mixture until the dye had penetrated the hair shaft and would not rinse off with water. Vibrissae were placed on a white background with the laterally flattened side down and held flat by a large glass slide to eliminate shape distortion. The camera was mounted on a photographic copy stand normal to the vibrissa and a remote shutter release was used to trigger imaging.

**Table 1:** Pinnipeds analyzed in this study.

Vibrissal Profile	Family	Species	Number of Individuals
Beaded	Phocidae	Gray seal (*Halichoerus grypus*)	8
	Phocidae	Harbor seal (*Phoca vitulina*)	15
	Phocidae	Harp seal (*Pagophilus groenlandicus*)	16
	Phocidae	Ringed seal (*Pusa hispida*)	16
	Phocidae	Spotted seal (*Phoca largha*)	9
Smooth	Phocidae	Bearded seal (*Erignathus barbatus*)	10
	Otariidae	California sea lion (*Zalophus californianus*)	12
	Otariidae	Guadalupe fur seal (*Arctocephalus townsendi*)	1
	Otariidae	Northern fur seal (*Callorhinus ursinus*)	2
	Otariidae	South American fur seal (*Arctocephalus australis*)	2
	Otariidae	Steller sea lion (*Eumetopias jubatus*)	1

Individuals are classified by family, species and vibrissal profile.

**Figure 1:**
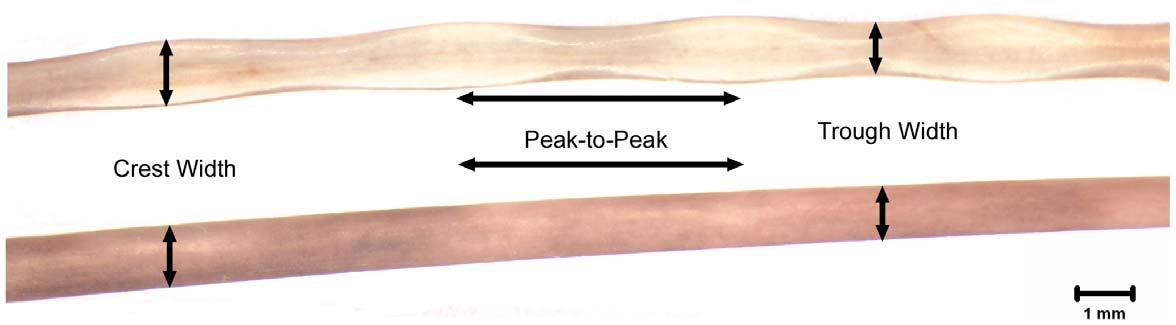
Locations of traditional morphometric measurements on beaded and smooth vibrissae. A ringed seal (top) and California sea lion (bottom) vibrissa are shown with locations of three traditional morphometric measurements (peak-to-peak distance, crest width and trough width) indicated by the black arrows. The measurements are labeled by the text between the two vibrissae. Peak-to-peak distance is not in reference to any physical structures on smooth vibrissae; it is simply the distance between successive measurements of vibrissal width.

Traditional morphometric measures were collected using the scaled digital photographs (27.0 pixels/mm) of whole vibrissae following Ginter et al. [17]. Traditional morphometric measurements collected were: peak-to-peak distance (linear distance between successive crests along the top and bottom margins of each vibrissa), crest width (width of beads), trough width (width of constrictions), and curvilinear length of the entire vibrissa (Fig. 1). All measurements were made using ImageJ (version 1.41, National Institutes of Health, Bethesda, MD, USA; http://rsbweb.nih.gov/ij/). Each of the five measurement types was made multiple times on each vibrissa. The number of measurements made on a vibrissa depended on the number of crests along the length. The mean number of crests along all beaded vibrissae was used as the number of measurements taken, distributed evenly along smooth vibrissae. Values were averaged for each individual vibrissa. The ratio of mean crest width to mean trough width was computed for each species. The geometric mean of all traditional morphometric measures for each individual was used as a standard proxy for overall size in statistical analysis of the traditional morphometrics [31]. Level A data (age class and gender) were obtained from the recovering stranding network for 45 individuals from five species.

**Figure 2:**
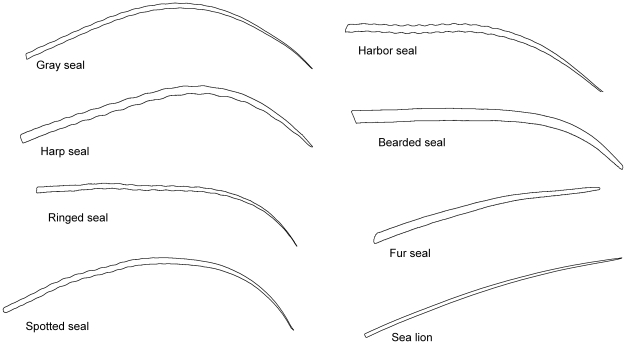
Example outlines of a vibrissa from each species analyzed. Left column from top to bottom: gray, harp, ringed and spotted. Right column from top to bottom: harbor, bearded, fur seal and sea lion.

For geometric morphometric characterization, we used outline-based, rather than landmark-based analysis due to the lack of homologous landmarks on vibrissae. The same images used for the traditional morphometric measurements were thresholded using ImageJ. TpsDig2 software [32] was used to fit an outline to each thresholded vibrissal image (Fig. 2), calculate the area within the outline, and save the series of X-Y coordinates for each outline. The coordinates were renumbered using a Microsoft Excel 2007 algorithm to standardize the location of “point 1” at the middle of the base of each vibrissa. The renumbered coordinates were imported to EFAWin software [33] for elliptic Fourier analysis (EFA). Harmonics were added sequentially until digital reconstructions of the vibrissae outlines were judged by eye to completely represent the outlines of all vibrissae in this study. Good fit was apparent with 15 harmonics. The shape variables that resulted were set to be invariant to size (area of the first harmonic ellipse), orientation, and rotation. Size of the first ellipse (area or length of the major axis) is a reasonable and accepted standard for calculating subsequent harmonic coefficients [33], [34]. However, area within the outline, measured in pixels using tpsDig2, was used as a more biologically relevant measure of size in our statistical analyses.

Elliptic Fourier harmonic coefficients and the log-transformed traditional morphometric measures were combined to create a more complete analysis of vibrissal form. However, since the two methods measured vibrissae in different units, the scale of each dataset was converted to match their true rank to be comparable. Matrix rank is the number of linearly independent columns or rows in a data matrix [35]. In a more practical vein, the true rank is the number of components that collectively summarizes variance above “noise” such as digitization error [36]. Such thresholds are usually set to encompass 95 or 99% of total variance. True rank scaling is accomplished by multiplying each data matrix by the square root of the ratio of the data's true rank divided by the original variance of the data matrix. Data scaled in this manner can be entered together into a single principal components decomposition (T. J. DeWitt, In prep.).

### Statistical Analyses

All statistical analyses used JMP software (version 8.0.1, SAS Institute, Inc., Cary, NC), with the exception of partial η^2^, which was calculated using SPSS (version 14.0.1, SPSS Inc.). Discriminant function analysis (DFA) provided a heuristic measure of how well each morphometric method performed in correctly classifying individual pinnipeds to species based on vibrissal shape. Quadratic, as opposed to linear, DFA allows for each taxon to be predicted based on its own variance-covariance matrix in the canonical space. Different covariance structures can result from factors such as small sample sizes (e.g., <30 individuals per taxon). We ran both types of DFA in each of the three analyses to provide a complete comparative basis for each method. Results of all statistical analyses were considered to be significant at p<0.05.

#### Traditional Morphometrics

All traditional morphometric data were log (x+1) transformed for normality. This transformation was used to avoid negative numbers that could not be used in calculation of geometric mean size. Species-specific differences in morphological variation were tested using multivariate analysis of variance (MANOVA) with species, geometric mean and an interaction effect between species and geometric mean as the independent variables and the log-transformed linear distances as the dependent variables. To remove the effect of size in the dataset before evaluating the species effect using DFA, a second MANOVA was run with only geometric mean as the independent variable and the log-transformed linear distances as the dependent variables. Residuals from that analysis were subsequently used in both linear and quadratic DFA with the species effect. One-Way ANOVA with Tukey HSD post-hoc tests was used to evaluate differences among species in morphometric measurements on the vibrissae. Such use of ANOVA following MANOVA is referred to as “protected” ANOVA, and is a common practice in ecology and evolutionary biology [37]. However, significance levels for these ANOVA tests should be taken less as precise statements and more as useful heuristic devices, to help distinguish which original variables contribute most strongly to differences between taxa in the multivariate space. These “protected” p-values are demarcated herein using the approximation symbol “≈”. A separate One-Way ANOVA was run to evaluate differences among age classes and between genders for vibrissal length and area.

#### Geometric Morphometrics

The EFA coefficients were compared among species using Principal Components Analysis (PCA) on correlations. Principal components (PCs) on correlations, rather than covariances, were used because small-scale differences in vibrissal morphology were more important in discriminating species than large-scale differences, such as overall curvature. PCs on covariances tend to weight large-scale differences more heavily, while PCs on correlations give all variables equal weight [38]. The number of principal components necessary to summarize 99% of the variation in the dataset was used in subsequent analyses. Area within the vibrissal outline was used as the measure of size for the geometric morphometric analysis. Since area is a squared (two dimensional) measurement, the square root of area was taken to convert this value to a linear measurement, which would be comparable to the linear traditional measurements. The square root of area was subsequently log transformed for normality. A MANOVA was run with species, log-transformed square root of vibrissae area and an interaction effect between species and vibrissae area as the independent variables and principal components of Fourier coefficients as the dependent variables. To remove the effect of size in the dataset before conducting DFA, a second MANOVA was run with only log-transformed square root of vibrissae area as the independent variable and principal components summarizing 99% of the variance as the dependent variables. Residuals from that analysis were used in both linear and quadratic DFA with the species effect.

#### Combined Traditional and Geometric Morphometric Data

To scale each of the morphometric datasets to its true rank, we used eigenvalues of the covariance matrix from the PCA for the traditional morphometric dataset and the correlation matrix from the PCA for the geometric morphometric dataset. True rank was defined as the number of principal components needed to summarize 99% of the variance in each dataset. Once scaled, the datasets were subsequently combined and covariance principal components were generated for the new data matrix. The principal components needed to summarize 99% of variance in the combined dataset were used as dependent variables in a MANOVA with species, geometric mean, log-transformed square root of vibrissae area, interaction effects between species and each size measure, an interaction effect between the two size measures and a three-way interaction effect between species and both size measures. To remove the effect of size in the dataset before DFA, a second MANOVA was run with only geometric mean and log-transformed square root of vibrissae area as the independent variables and principal components as the dependent variables. Residuals from that analysis were used in linear and quadratic DFA with the species effect.

## Results

### Traditional Morphometrics

To maintain the comparative aspect of the study, we measured smooth vibrissae, which do not have crests and troughs, at approximate points analogous to the crest and trough locations on beaded vibrissae (Fig. 1). The mean peak-to-peak distance measured along the top and bottom margins of all beaded vibrissae was used as a guide to measure width at multiple locations on smooth vibrissae. These width measurements were analogous to crest width on beaded vibrissae. Since smooth vibrissae do not have a sinusoidal profile (i.e., changes in width along the length of the vibrissa), our calculation of the ratio of crest width to trough width was 1 by definition for otariids and bearded seals (Table 2). Although we refer to width values of smooth vibrissae as being crest and trough widths, this nomenclature was used simply to enable comparison with beaded vibrissae and does not describe the shape of smooth vibrissae.

**Table 2:** Results of traditional and geometric morphometric measurements.

	Top Peak-to-Peak Distance (mm)	Bottom Peak-to-Peak Distance (mm)	Crest Width (mm)	Trough Width (mm)	Crest Width/Trough Width	Total Length (mm)	# of Beads/cm
Harp	3.97±0.70	3.94±0.69	0.88±0.14	0.69±0.13	1.28	80.6±10.8^ab^	2.2±0.4^bc^
Harbor	3.27±0.39	3.26±0.40	0.92±0.13	0.73±0.12	1.26	86.5±10.5^ab^	2.3±0.4^abc^
Ringed	3.56±0.73	3.53±0.72	0.70±0.21	0.49±0.21	1.44	79.6±10.9^ab^	2.5±0.5^ab^
Spotted	4.01±0.63	3.99±0.63	1.04±0.15	0.85±0.15	1.22	91.2±7.9^a^	1.9±0.2^c^
Gray	3.43±0.38	3.41±0.39	0.76±0.13	0.63±0.11	1.21	73.8±12.5^b^	2.7±0.5^a^
Bearded	3.64±0.66	3.62±0.65	1.18±0.27	1.18±0.27	0.99	77.2±9.5^ab^	0
Fur Seals	3.64±0.66	3.62±0.65	0.96±0.12	0.96±0.12	1.01	83.0±20.3^ab^	0
Sea Lions	3.64±0.66	3.62±0.65	0.84±0.19	0.84±0.19	1.00	90.7±11.3^a^	0

**Figure 3:**
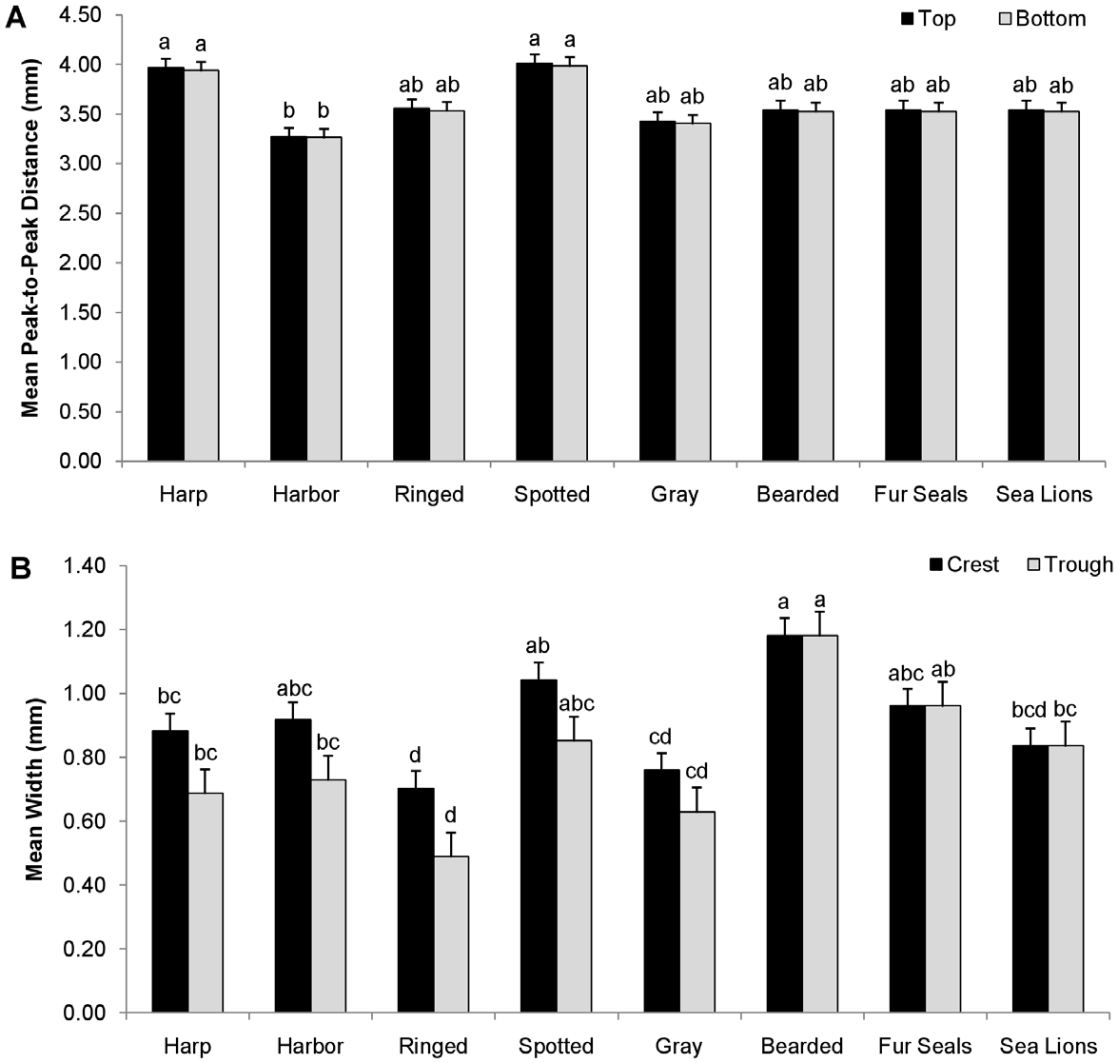
Results of the traditional morphometric measurements. Vibrissae from five species of phocid seals with beaded vibrissae, one species of phocid with smooth vibrissae (bearded) and otariids were analyzed. One-Way ANOVA's were performed following overall significance of species effects in MANOVA. Species with the same letter were not significantly different; species with different letters were significantly different for the given measure (Tukey HSD; p<0.05). A) Mean (+SE) peak-to-peak distances along the top and bottom margins of the vibrissa. B) Mean (+SE) crest and trough widths. Since smooth vibrissae do not have crests and troughs, both measurements simply describe the vibrissal width.

Mean ± SD values for each species are given. Significant differences between species for total length and number of beads per cm are indicated by different letters. Significant differences between species for the other traditional morphometric measurements are given in Figure 3. Species with different letters are significantly different from one another.

The total length of vibrissae analyzed in this study ranged from 60 mm to 110 mm. The number of beads per cm along the vibrissa ranged from 1.1 (harp seal) to 3.4 (gray seal) and species were significantly different in this variable (p≈0.002; Table 2). For beaded vibrissae, the mean values for the peak-to-peak distance along the top and bottom vibrissal margins were nearly identical, indicating that the distance between successive crests is not affected by curvature of the overall vibrissa (Table 2, Fig. 3A). Bearded seals had the widest smooth vibrissae and spotted seals (*Phoca largha*) showed the greatest mean crest and trough widths of all beaded vibrissae. The lowest crest and trough widths were seen in ringed seals (*Pusa hispida*; Table 2, Fig. 3B). However, ringed seals had the highest crest width to trough width ratio, indicating that these vibrissae have the most pronounced sinusoidal profile of the five phocid species investigated with beaded vibrissae. Spotted seals had the lowest crest width to trough width ratio, indicating that these seals have the least pronounced sinusoidal profile of the five beaded phocid species. Crest width to trough width ratios of harp (*Pagophilus groenlandicus*) and harbor (*Phoca vitulina*) seals were nearly identical to each other as were the crest width to trough width ratios of spotted and gray (*Halichoerus grypus*) seals (Table 2).

**Figure 4:**
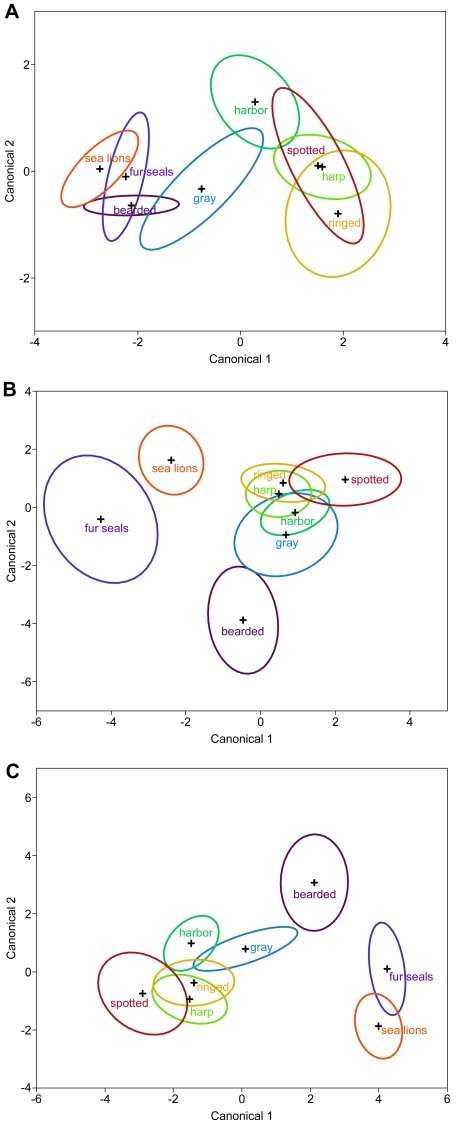
Centroid plots for the quadratic discriminant function analyses (QDFA) on the three morphometric methodologies. Crosses mark the mean for each species; ellipses are 95% confidence regions. A) Results of QDFA on the traditional morphometric measures. B) Results of QDFA on elliptic Fourier harmonic coefficients (geometric morphometrics). C) Results of QDFA on the combined traditional and geometric morphometrics.

Quadratic DFA on the traditional morphometric measures separated beaded species from non-beaded species on Canonical axis 1 (Fig. 4A). In this analysis otariids separated into two groups that slightly overlapped with one another. Bearded seals (smooth vibrissae) overlapped with both gray seals from the beaded phocid cluster and fur seals in the otariid group. Gray seals overlapped only with harbor seals from the beaded phocid cluster. Spotted and harp seals overlapped extensively, while ringed and harbor seals were completely separated from each other. Canonical axis 2 was likely composed of more than one variable and may have separated species primarily based on overall vibrissal length, with harbor seals being further separated from the beaded cluster by their lower peak-to-peak distances (Table 2). Interestingly, gray seals had the highest mean number of beads per cm but were located closer to the smooth-whiskered species in morphospace (Fig. 4A). Quadratic DFA showed that the traditional morphometric measures correctly classified 73 out of 92 individuals (79.3%; Table 3).

**Table 3:** Results of the MANOVA for the three analyses.

		F	DF	p	Partial η^2^	LDFA %	QDFA %
Traditional	Species	9.2	35,305.3	<0.001	0.45	64.1	79.3
	Geomsize	6867.5	5,72	<0.001			
	Species*Geomsize	3.1	35,305.3	<0.001			
Geometric	Species	2.5	126,397.7	<0.001	0.42	78.3	97.8
	LogSqrtArea	3.4	18,59	<0.001			
	Species*LogSqrtArea	1.7	126,397.7	<0.001			
Combined	Species	4.4	133,340.9	<0.001	0.50	84.8	100.0
	LogSqrtArea	10.1	19,50	<0.001			
	Geomsize	8.9	19,50	<0.001			
	Species*LogSqrtArea	2.8	133,340.9	<0.001			
	Species*Geomsize	2.3	133,340.9	<0.001			

Partial η^2^ is a measure of effect strength. LDFA% and QDFA% are the percentages of individuals that were correctly classified by the linear discriminant function analysis and quadratic discriminant function analysis, respectively. Geomsize (geometric mean) and LogSqrtArea (log of the square root of the area) were the measures of vibrissal size.

There were significant differences between some species in each of the traditional morphometric measurements. ANOVA with Tukey post-hoc results showed that harp and spotted seals significantly differed from harbor seals (p≈0.004 and p≈0.010, respectively; Fig. 3A) for the log of peak-to-peak distance along the top margin of the vibrissa. The other species did not significantly differ from one another for this variable. Harp and spotted seals also significantly differed from harbor seals (p≈0.005 and p≈0.013, respectively) for the log of peak-to-peak distance along the bottom margin of the vibrissa, and the other species did not significantly differ from one another in this variable (Fig. 3A). Bearded seals differed from all species except spotted (p≈0.964), harbor (p≈0.144) and fur seals (p≈0.809) for the crest width variable, and ringed seals differed from all species except sea lions (p≈0.258) and gray seals (p≈0.948; Fig. 3B). Ringed seals differed from all species except gray seals for the trough width variable (p≈0.066), and bearded seals differed from all species except fur (p≈0.835) and spotted seals (p≈0.085; Fig. 3B). Again, bearded seals, fur seals and sea lions do not have physical crests and troughs so these comparisons refer to their vibrissal width values at points analogous to crests and troughs on beaded vibrissae (Fig. 1). Species were not significantly different in the log length of the vibrissae variable, except for gray seals, which differed from sea lions and spotted seals (p≈0.021 and p≈0.029, respectively; Table 2). Intraspecific variation could not be effectively assessed due to the low number of individuals per species.

Individual animals were classified by the recovering stranding network or indigenous group into one of five age classes: pup, yearling, juvenile, subadult or adult. The five age classes did not have significantly different vibrissal lengths (p = 0.058) or vibrissal areas (p = 0.144). Additionally, males and females did not differ in vibrissal length (p = 0.9169) or vibrissal area (p = 0.825).

### Geometric Morphometrics

A good (identical by eye) outline fit of each vibrissa was obtained with 15 harmonics. Four Fourier coefficients (A, B, C, D) were generated to describe each of the 15 harmonics, plus the two zeroth (A0 and C0) harmonics, for a total of 62 shape variables. Fifteen harmonics provided enough variability in shape definitions that 18 PCs summarized 99% of the variance within the dataset. We therefore used these 18 variables as our metrics of shape in subsequent analyses. Both linear and quadratic DFA provided good discrimination among species based on shape (Table 3). Due to the greater discriminatory ability of quadratic DFA we focused on those results. The quadratic DFA on the geometric morphometric measures separated otariids from phocids (Fig. 4B). The phocids with beaded vibrissae clustered together and all species overlapped with each other. Additionally, the otariids separated into two non-overlapping groups, whereas these same groups overlapped slightly in the traditional plot. Bearded seals were positioned between beaded phocids and otariids, but were completely separated from the phocid cluster and both otariid groups, in contrast to the traditional plot. Canonical axis 1 appeared to separate beaded vibrissal species from non-beaded vibrissal species, as seen in the traditional plot. Canonical axis 2 may have separated species based on overall vibrissal length, since sea lions and bearded seals had the longest and shortest smooth vibrissae, respectively, and spotted and gray seals had the longest and shortest beaded vibrissae, respectively (Table 2). Alternatively, this axis may be detecting differences in cross-sectional shape, or an interaction of several shape characteristics. Subsequent canonical axes made only minor contributions to discriminatory ability. Quadratic DFA showed that the principal components of elliptic Fourier harmonic coefficients facilitated classification of 90 out of 92 individuals (97.8%; Table 3).

### Combined Traditional and Geometric Morphometric Data

The traditional and geometric morphometric data exhibited shared and unique aspects of discriminatory ability. The redundancy (similar taxonomic discrimination) implies a combined analysis is more appropriate, more complete and more powerful. A PCA on the EFA harmonic coefficients showed that 18 PCs were required to summarize 99% of the variance in the dataset compared to a PCA on the traditional dataset, which only needed three PCs to summarize 99% of the variance in the data. Therefore, the true ranks of the geometric and traditional morphometric datasets were 18 and three, respectively. If there were no redundancy between the two datasets, a combined, expected 21 PCs would be required to summarize 99% of the variance in the dataset. However, the combined dataset required 19 PCs on correlations to summarize 99% of the variance in the dataset. This showed that there was some overlap between the traditional and geometric morphometric methodologies. The MANOVA results for the combined data are summarized in Table 3. Since two different measures of size were used in the traditional and geometric morphometric analyses, both size measures were included in the combined data model. Non-significant interaction effects were removed from the model.

Quadratic DFA again clearly separated phocids and otariids on Canonical axis 1 with bearded seals occupying an intermediate position between otariids and beaded phocids (Fig. 4C). Canonical axis 2 again appeared to separate species in our dataset based on vibrissal length. This separation was clear for the smooth-whiskered species, but the small-scale intricacies of the beaded profile likely complicated the relationships between the beaded phocids. For example, spotted seals had the longest vibrissae but may have been pulled along Canonical axis 2 towards harp seals and away from harbor seals by the peak-to-peak distance variables (Table 2). Surprisingly, gray seals, with the highest mean number of beads per cm, were again pulled towards the smooth-whiskered species. In this analysis, harbor and gray seals did not overlap with either spotted or harp seals, but did overlap with each other and ringed seals. Harp and ringed seals overlapped considerably (Fig. 4C). In contrast to the centroid plots for the geometric and traditional morphometrics, otariids loaded higher than phocids on Canonical axis 1 and bearded seals had the highest loading on Canonical axis 2.

Quadratic DFA on the combined dataset correctly classified 100% of individuals (Table 3), compared to 79.3% and 97.8% for the traditional and geometric datasets, respectively. This demonstrated that each method found differences between species that the other method did not. The combined methodology incorporated all differences and was the best at correctly classifying species. In all three analyses, quadratic DFA outperformed linear DFA in correctly classifying individuals (Table 3). Since all factors in the statistical models were significant, we compared the proportion of partial variance explained by the main effect of interest, species, using Wilks' partial η^2^. This value was similar for all three analyses but highest for the combined dataset (Table 3). This indicates that the species effect was relatively strongest in the combined analysis, and relatively weakest in the geometric morphometric analysis. However, the species effect still explained approximately 50% of the variance in each of the three models.

## Discussion

Phocids with beaded vibrissae show species-specific variation on a common sinusoidal beaded pattern. In all analyses, the phocids possessing beaded vibrissae clustered together. However, it is interesting to note that the only two congeneric phocids in the study, harbor and spotted seals, did not overlap at all in the combined data analysis and overlapped only partially in the geometric morphometric and traditional morphometric analyses. Gray seals appeared to occupy an intermediate position in morphospace between the other beaded phocids and smooth-whiskered bearded seals in all three analyses. This was surprising since this species had the highest number of beads per cm and the overall shape appeared quite similar to the rest of the beaded phocids. Ginter et al. [17] initially found a different pattern in traditional morphometric measurements along the vibrissae of gray seals compared to harp and hooded (*Cystophora cristata*) seals, but that difference was not maintained when additional samples were added (C.C. Ginter, unpubl. data). Gray seals possess different head morphology from other phocids. Cameron [39] and King [11] described the nose of male gray seals as high and arched, while females have a long, straight profile to the top of the head. Gray seals are similar to hooded and elephant (*Mirounga* spp.) seals in that the males have enlarged snouts used in visual signaling [40]. The broader snout may change the position of vibrissae on the muzzle. As a result of this difference in location, vibrissae may have evolved an alternative morphology. While the phocid species with beaded vibrissae always clustered together in morphospace, the fact that all individuals could be correctly classified to the species level demonstrated that the beaded profile is not identical. We sampled a large number of Phocinae species, but not all members of this subfamily were included in the study. However, based on the variation within this beaded vibrissal group, we predict that the rest of family Phocidae would cluster with the beaded phocids examined here.

Bearded seals, a phocid with smooth vibrissae, were positioned between beaded phocids and otariids in morphospace. Interestingly, the centroid ellipses for bearded seals and otariids (which also possess smooth vibrissae) never overlapped in the geometric morphometric or the combined data analysis. In fact, the centroid ellipse for bearded seals was closer in morphospace to the beaded phocids than to the smooth-whiskered otariids in the geometric and combined analyses. This strongly suggests that the smooth vibrissae of bearded seals are different from the smooth vibrissae of otariids in this study. This difference may be related to cross-sectional shape. Bearded seal vibrissae are almost rectangular in cross-section and this shape differs considerably from the oval cross-sectional shape of otariids and other phocids ([16], pers. obs.). A limitation of this comparative analysis is that the other phocids with smooth vibrissae, monk seals (*Monachus* spp.), were not included. However, based on personal observations and diet studies in the literature, we predict that monk seals would not occupy the same morphospace as bearded seals. Rather, they may be intermediate between bearded seals and the beaded phocid cluster or intermediate between bearded seals and otariids. The differences among pinniped vibrissae without a beaded profile were a surprising result of this study. We have reported evidence that there is variation in smooth vibrissal shape and morphology among otariids, since the geometric morphometric approach completely separated otariids into two groups, and the combined data analysis demonstrated only a minimal overlap between these groups. Clearly these differences in morphology and shape previously have been overlooked and may have important ecological consequences.

It is important to note that although the comparisons of vibrissal length and area between genders and among age classes are interesting, full Level A data were only obtained for 45 out of 92 individuals from five species. More males than females were obtained (34 males versus 16 females) and there were more known adult males than known adult females, both of which may have biased the results. However, additional support for the observed lack of difference in vibrissal length among age classes comes from Scheffer's [41] observation of a full term Northern fur seal (*Callorhinus ursinus*) fetus with vibrissae as long as 63 mm and Bonner's [10] observation of a three week old Antarctic fur seal (*Arctocephalus gazella*) with vibrissae up to about 80 mm long. A four and a half month old Southern elephant seal (*Mirounga leonina*) fetus had mystacial vibrissae up to 27 mm long [27]. A ringed seal pup of the year in the present study had vibrissae that were slightly longer than the mean value for that species. Although caveats are certain to arise, the data analyzed here suggest that there are no gender or age class effects on vibrissal morphology and shape.

Although we focus on the shape of individual vibrissae here, other factors also are likely to be important in vibrotactile sensory perception, such as location, distribution of the vibrissae on the muzzle, and innervation. The function of the entire mystacial vibrissa pad is most likely an interaction between vibrissal hair shaft shape, the geometry and location of the vibrissae. The interaction of morphology at these two scales is likely to be related to foraging mode and strategy. Bearded seals and walruses exemplify the importance of the geometry of mystacial vibrissae location. The distribution of bearded seal vibrissae differs from other phocids. Instead of lying along the lateral sides of the rostrum, bearded seals have vibrissae widely distributed over the anterior portion of a blunt muscular muzzle [9], [16]. Bearded seals forage for benthic invertebrates [42]. Walruses, another benthic foraging specialist, exhibit a similar vibrissal distribution [43]. This vibrissal arrangement is related to a benthic foraging mode and appears to be convergent with the vibrissal arrangement on the oral disk of sirenians, which also spend considerable time foraging on the benthos [44]–[46]. Due to the orientation of bearded seal and walrus vibrissae [16] it is unlikely that these benthic foragers are protracting the vibrissae into the flow field, and therefore their function will be different than either beaded or other smooth vibrissae.

Otariids have smooth vibrissae and feed on similar prey to phocids with beaded vibrissae [47], which suggests that beaded vibrissae are not critical in catching certain prey items. However, otariids generally do not dive to the depths that phocids do in search of prey. The greater amount of ambient light present in shallower water may allow otariids to rely more heavily upon vision for prey detection and capture or a combination of visual, auditory and tactile cues [48]. Other ecological factors such as time of day of foraging, actively swimming versus sedentary prey, foraging on the bottom versus in the water column and water turbidity may also affect the presence of beading along pinniped vibrissae. Both California sea lions and harbor seals are able to detect water velocities below those that would be generated by a swimming fish using their vibrissae [19], [49]. However, blindfolded California sea lions could successfully track a hydrodynamic trail using their vibrissae only 50% of the time when the signal made a single turn. There was also a decrease in tracking ability when there was a delay of more than a few seconds between the generation of the trail and the beginning of the sea lion's search for it [48]. The animal always failed to find the hydrodynamic trail when the vibrissae were covered by a stocking mask [48]. These performance data suggest that vibrissae are an important sensory modality in this species, but are not the only sensory system involved in prey tracking, since it is unlikely that a chased fish will swim in a straight line.

In contrast, harbor seals are able to follow a complex hydrodynamic trail as long as 40 m with high accuracy, even with glide phases in the trail, and can determine the direction of a trail after delays up to 35 s [20], [24], [25]. Additionally, these seals are able to follow a trail, even when they contact it at an obtuse angle, by repeatedly crossing the trail and gradually narrowing the angle. Such a search method would be more successful in tracking fleeing fish [23]. As seen in California sea lions, harbor seals never found the hydrodynamic trail when the vibrissae were covered with a stocking mask [20], [23]–[25]. The beaded profile of harbor seals' vibrissae was shown to suppress self-induced vibrations caused by ambient water flow during swimming [30]. It is likely that reduced vibrations of the vibrissae allow detection of hydrodynamic trails as prey turn away from the seal during escape maneuvers. Objects of different sizes and shapes can be perceived based on characteristics of their resulting hydrodynamic trail by harbor seals' vibrissae [50]. Since we have shown that morphology and shape of beaded phocid vibrissae are species-specific variants on a basic pattern, the performance data from harbor seals may not be completely representative of all phocids with beaded vibrissae.

The different sizes and shapes of beaded vibrissae may have functional consequences related to identifying the hydrodynamic signal of prey that have not yet been explored. Different shaped paddles created different flow fields that were quantified using digital particle imaging velocimetry [50]. The species-specific differences observed in beaded vibrissae may be related to subtle changes in the flow fields created by prey of slightly different size or shape. However, this possibility would best be explored using live pinnipeds with different vibrissal morphologies chasing live prey that create different flow fields. The biological mechanisms responsible for beading and species-specific differences in beaded morphologies are currently not understood. One possible mechanism affecting beading is periodic variation in keratin deposition rate during vibrissal growth. Regardless of the process, the resulting shapes likely relate to functional differences that should be investigated in live animals.

In summary, although the classic view is that pinniped vibrissae exhibit two distinct vibrissal morphologies, beaded and smooth, the morphology and shape of pinniped vibrissae within this study fall into at least three distinct groups: phocids with beaded vibrissae, phocids with smooth vibrissae, and otariids. A fourth group may be identified if additional research substantiates (with additional species and greater sample size) the division of otariid vibrissal shape into two groups as shown in our analyses. Future research should investigate further the shape differences among smooth vibrissae of otariids and phocids. Behavioral performance data for additional phocids and otariids will help elucidate the potential functional and ecological diversification that correlates with the variation in vibrissal morphology and shape reported in this study. Finally we hope to highlight the methodological insight that geometric and traditional morphometrics should not be treated as alternatives. It is fashionable to compete the two approaches to see which is “best” (e.g., [51], [52]). Rather, the two types of analysis should generally be used in harmony, by fusing the data as illustrated herein, to yield the most complete understanding of morphology. In the present case, 100% of vibrissae could be classified to taxon, which is a testament to both the synthetic methodology and the biological diversity in vibrissal shape.
